# Supplementary material for: Meso- and Rac-[bis(3-phenyl-6-tert-butylinden-1-yl)dimethylsilyl]zirconium Dichloride: Precatalysts for the Production of Differentiated Polyethylene Products with Enhanced Properties
Source: Polymers (Basel). 2022 May 30;14(11):2217. doi: 10.3390/polym14112217 (PMC9183169; doi:10.3390/polym14112217)
Supplement: Supplementary file 1 [file polymers-14-02217-s001.zip › polymers-1748500-supplementary.pdf]

Contents

Synthesis of the metallocenes........S2

Figure S1. ^{1}H (400 MHz, top) and $^{13}C\{^{1}H\}$ (100 MHz, bottom) NMR spectra (CD_2Cl_2, 25 °C) of isolated *meso*-**2**........S3

Figure S2. ^{1}H (400 MHz, top) and $^{13}C\{^{1}H\}$ (400 MHz, bottom) NMR spectra CD_2Cl_2, 25 °C) of the *rac*-enriched complex **2**........S4

Figure S3. APPI+-MS spectrum of rac-**2**........S4

Attempted *racemo*- and *meso*-selective metallocene synthesis........S4

Attempted *racemo*-selective synthesis of 2........S6

Scheme S1. Attempted *racemo*-selective synthesis of complex **2** with *tert*-butylamido directing ligand. S6

Figure S4. ^{1}H NMR spectrum (CD_2Cl_2, 400 MHz, 25 °C) of the crude reaction mixture indicating *rac*-**3** as the major product........S7

Figure S5. ^{1}H NMR spectrum (400 MHz, CD_2Cl_2, 25 °C) of the crude reaction mixture between **3** and Me_2SiCl_2 affording complex **2** in ca. 4:1 *rac/meso* ratio........S7

Synthesis of [bis(3-phenyl-6-tert-butylinden-1-yl)dimethylsilyl]chlorozirconium tert-butyl amide.........S7

Reaction of Me_2SiCl_2 with [bis(3-phenyl-6-tert-butylinden-1-yl)dimethylsilyl]chlorozirconium tert-butyl amide. S8

Attempts towards the *meso*-selective synthesis of 2........S9

Scheme S2. Attempts towards the *meso*-selective synthesis of complex **2** [2]........S9

Figure S6. Aromatic region of the ^{1}H NMR spectrum (400 MHz, CD_2Cl_2, 25 °C) of the crude reaction mixture resulting from the attempted *meso*-selective synthesis of complex **2**, indicating the presence of both *rac*- and *meso* isomers (*ca*. 1:1 ratio) as well as unreacted ligand **1**........S10

*Synthesis of Li_2-**1** (Solution A)*........S10

Synthesis of $ZrCl_4(THF)_2$ (Solution B)........S10

Synthesis of Li-OAd (Solution C)........S10

Synthesis of $Zr(OAd)Cl_3$ (Solution D).........S10

*Synthesis of complex **4** and regeneration of dichloride species **2***........S10

PE samples characterization........S12

Figure S7. $^{13}C\{^{1}H\}$ NMR spectrum (100 MHz, TCB/C_6D_6, 135 °C) of a PE synthesised with *meso*-**2** in the absence of 1-hexene (Table 1, entry 1)........S12

Figure S9. GPC traces of the PEs made with **supp-*rac*-2** (orange) and **supp-*meso*-2** (blue) (Table 2, entries 1 and 2)........S13

Figure S10. GPC traces of the PEs prepared with **supp-*meso*-2** in the presence of difference amounts of 1-hexene (Table 2, entries 3, 5, 7 and 9): all curves are perfectly overlapped. S13

Computational Studies. S14

Cartesian coordinates S15

References.. S17

Synthesis of the metallocenes

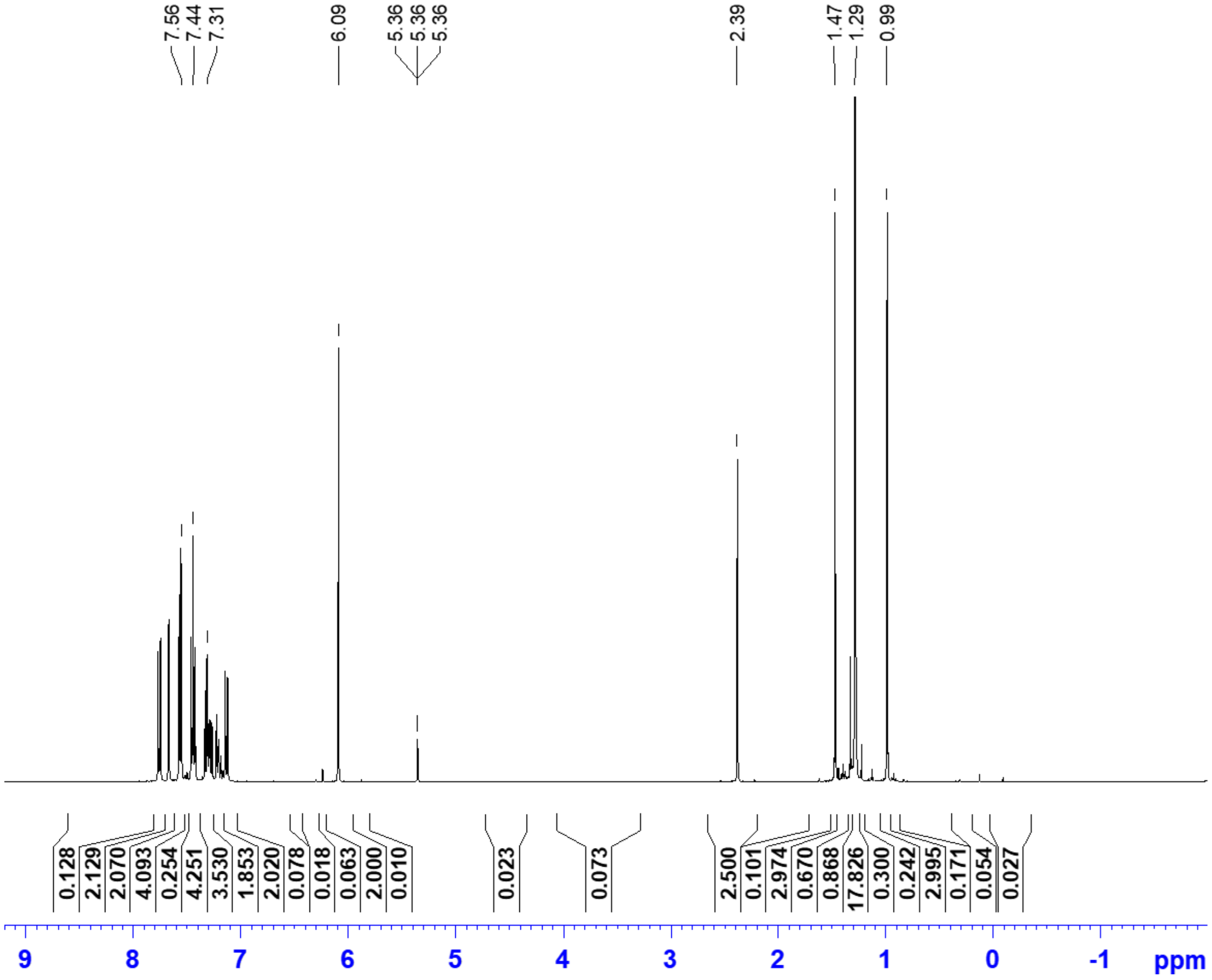

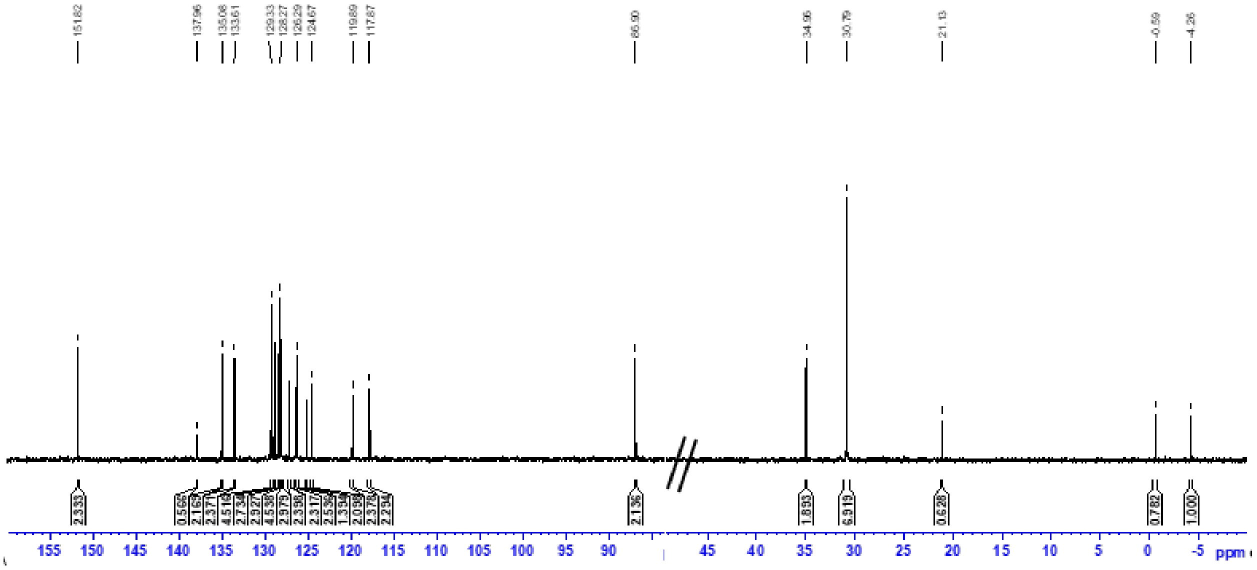

Figure S1. ^{1}H (400 MHz, top) and ^{13}C{^{1}H} (100 MHz, bottom) NMR spectra (CD_2Cl_2, 25 °C) of isolated *meso*-**2**.

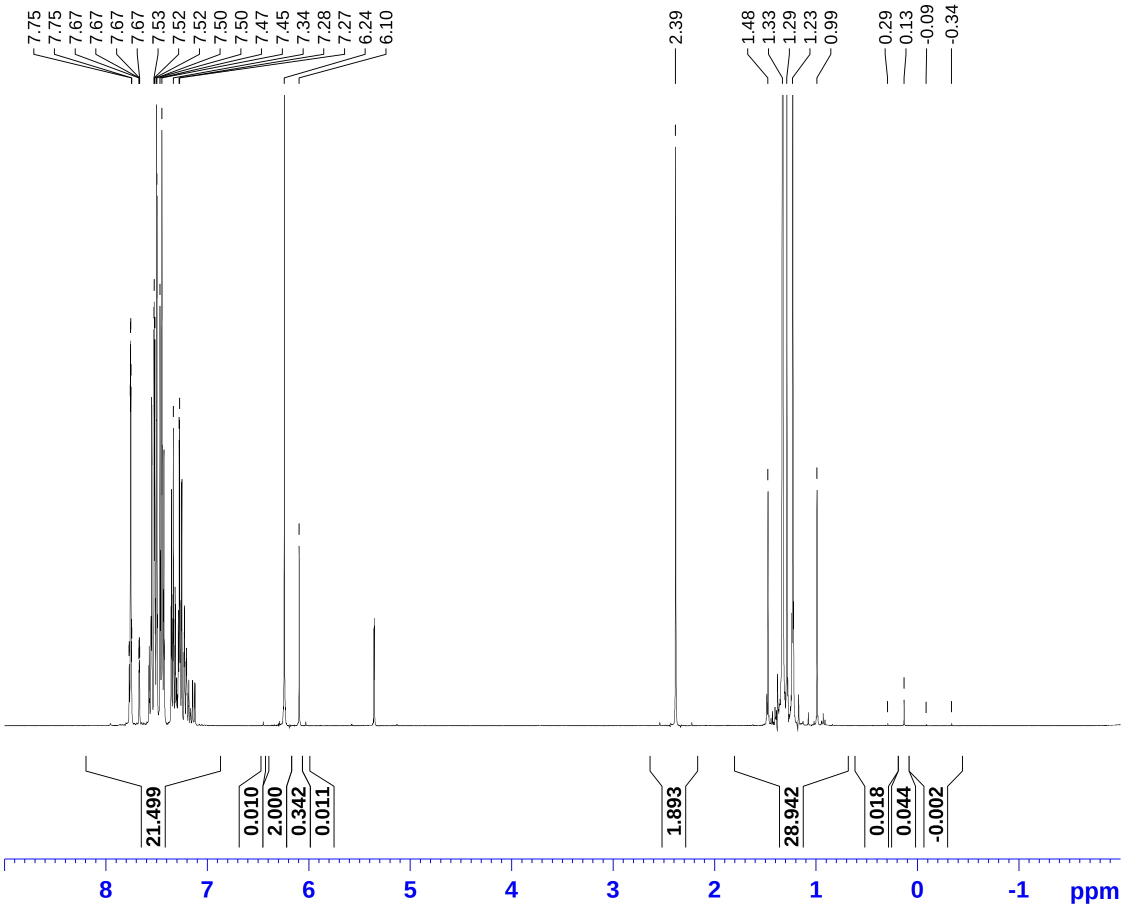

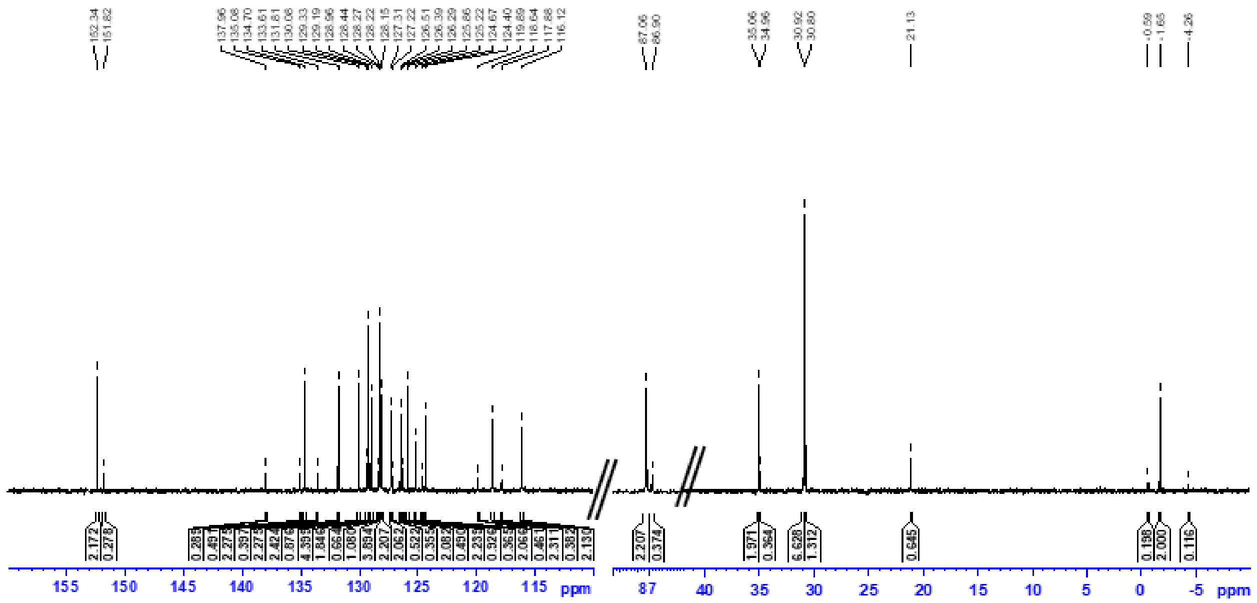

Figure S2. ^{1}H (400 MHz, top) and $^{13}C\{^{1}H\}$ (400 MHz, bottom) NMR spectra CD_2Cl_2, 25 °C) of the *rac*-enriched complex **2**.

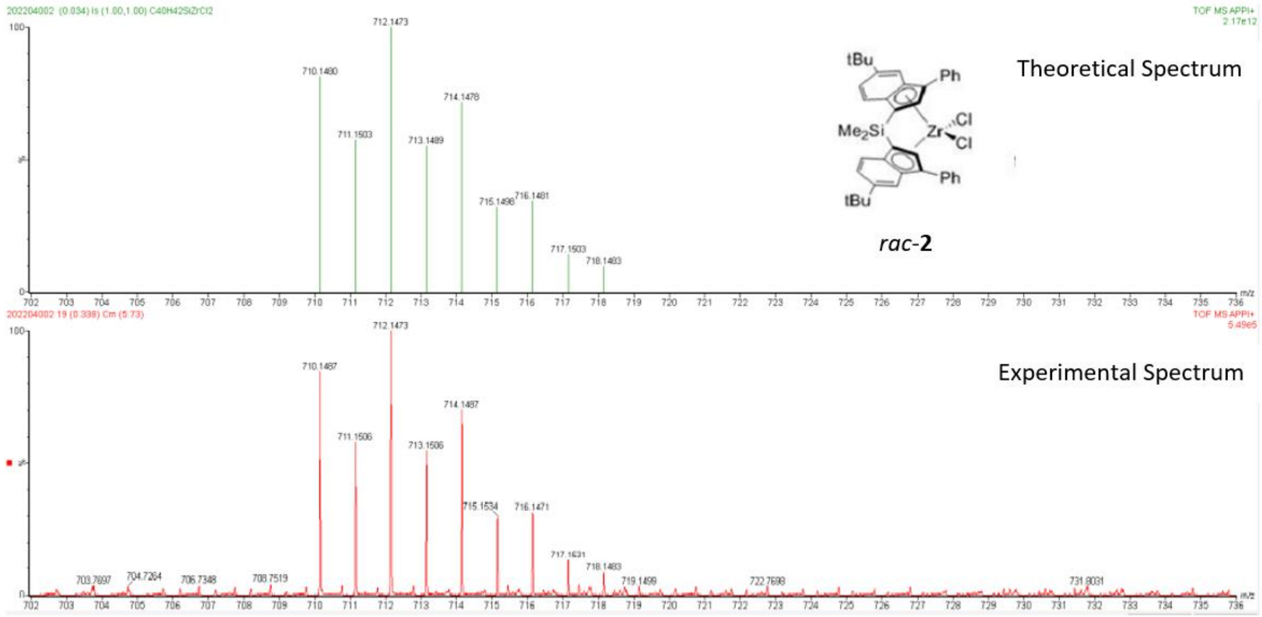

Figure S3. APPI+-MS spectrum of rac-**2**.

Attempted *racemo*- and *meso*-selective metallocene synthesis

Although the multiple crystallizations from toluene proved to be a viable method for the separation of *rac*- and *meso*-**2** isomers, the development of synthetic protocols allowing for the *preferential*

formation of one of the metallocene isomers is highly desirable. In this scenario, the use of a directing ligand has proven to be the most straightforward and effective approach towards *racemo*- [1] and *meso*-selective [2] bis(indenyl)-based metallocene synthesis.

Attempted *racemo*-selective synthesis of 2

A procedure analogous to that reported by Churakov [1] was used for the synthesis of NH*t*Bu-coordinated complex **3** (Scheme S1).

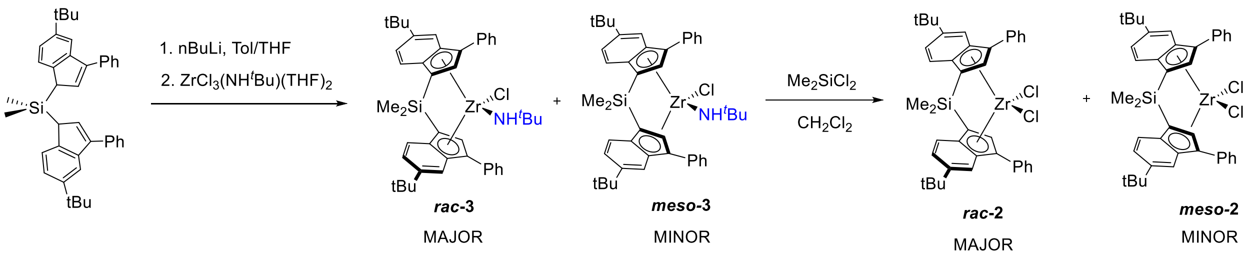

Scheme S1. Attempted *racemo*-selective synthesis of complex **2** with *tert*-butylamido directing ligand.

^{1}H NMR spectroscopy analysis of the crude product after metalation revealed a *ca.* 4:1 ratio of *rac:meso* metallocene **3** (Figure S4).

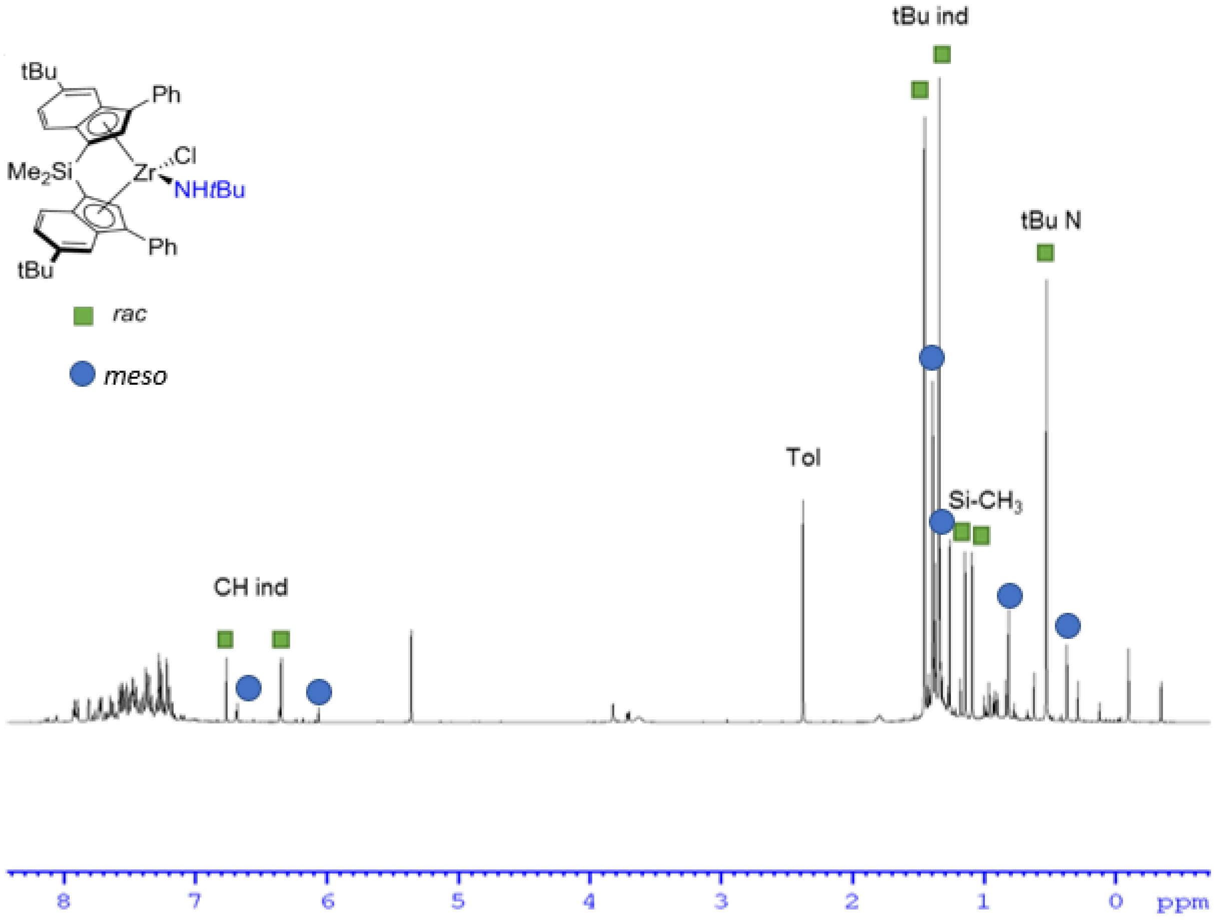

Figure S4. ^{1}H NMR spectrum (CD_2Cl_2, 400 MHz, 25 °C) of the crude reaction mixture indicating *rac*-**3** as the major product.

Treatment of such mixture with an excess of Me_2SiCl_2 afforded the dichlorozirconium metallocene **2**, retaining the same 4:1 *rac*/*meso* selectivity in the final product (Figure S5).

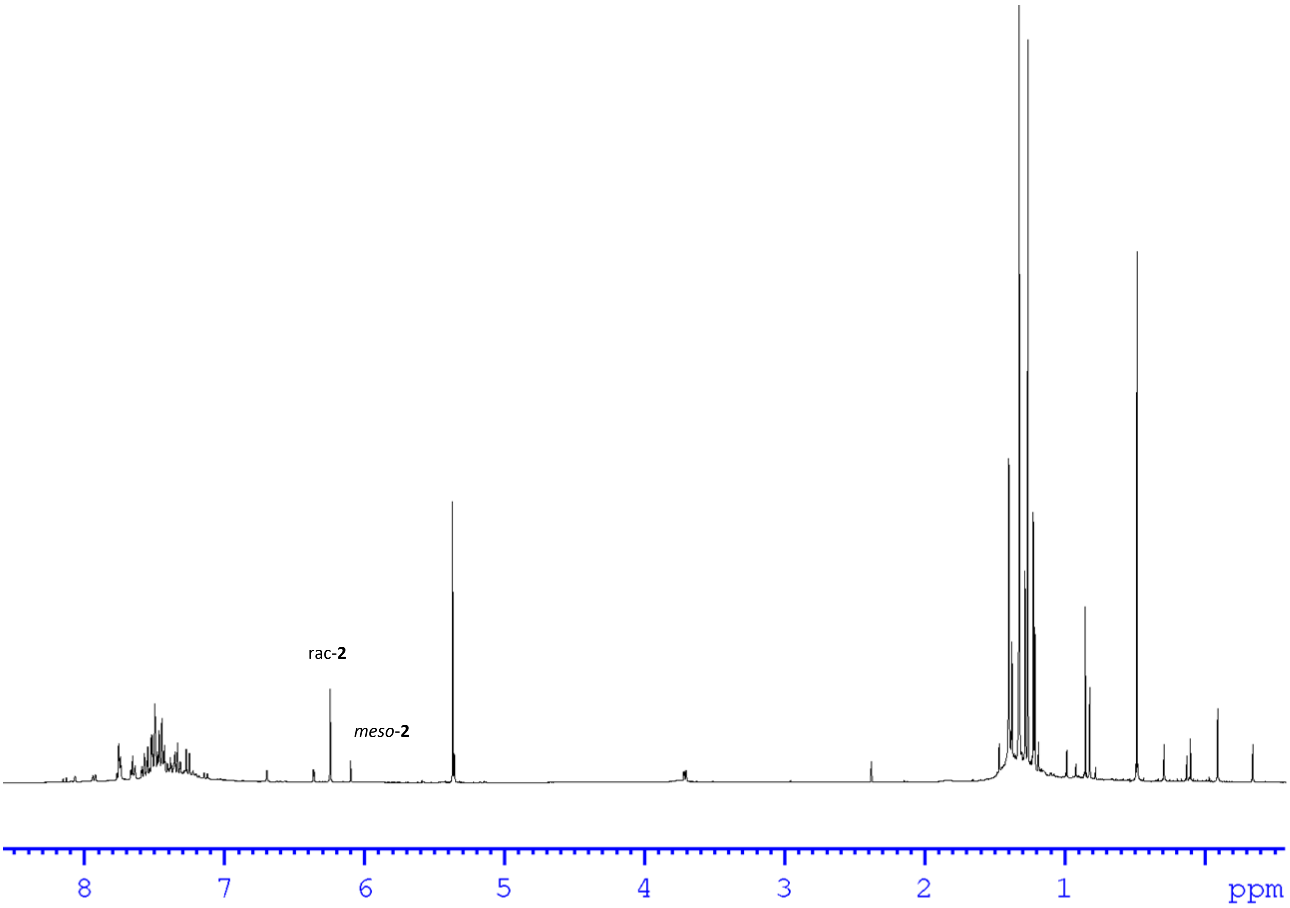

Figure S5. ^{1}H NMR spectrum (400 MHz, CD_2Cl_2, 25 °C) of the crude reaction mixture between **3** and Me_2SiCl_2 affording complex **2** in ca. 4:1 *rac*/*meso* ratio.

Synthesis of [bis(3-phenyl-6-tert-butylinden-1-yl)dimethylsilyl]chlorozirconium tert-butyl amide.

In the glovebox, *tert*-butylamine (70 mg, 0.95 mmol) was dissolved in a toluene/THF solution (ca. 10:1, 1.5 mL) in a 50-mL round bottom flask. To this solution *n*-BuLi (1.58 mL of a 1.6 M solution in hexanes, 0.99 mmol) was added dropwise at room temperature. An immediate colour change to

clear bright yellow occurred. The reaction mixture was left to stir for 16 h. In a separate flask, THF (171 mg, 2.37 mmol) was added dropwise to $ZrCl_4$ (222 mg, 0.95 mmol) suspended in toluene (3 mL), and was left to stir at room temperature for 1 h. A white solid suspension formed over the course of the reaction. The lithium *tert*-butyl amide reaction mixture was added dropwise to the $ZrCl_4(THF)_2$ suspension and left to stir at room temperature for 3 h (appearance: white solid with beige supernatant). Another solution with proligand **1** (526 mg, 0.95 mmol) dissolved in a toluene/THF mixture (10.4/1.4 *w/w*) was prepared (clear orange solution). To this solution, *n*BuLi (1.25 mL of a 1.6 M solution in hexanes, 2.0 mmol) was added dropwise at room temperature and the reaction mixture was left to stir for 2 h. To this mixture was added dropwise the trichlorozirconium *tert*-butyl amide reaction mixture. The colour changed to a dark orange suspension over the course of the addition. After 16 h, the mixture was filtered over a plug of Celite. The dark red filtrate was concentrated/dried *in vacuo* affording a red-orange glassy solid. Crude product yield: 318 mg (45% yield, ca. 75% purity). A ^{1}H NMR spectrum of the crude product (Figure S4) revealed that the *rac* isomer of NHtBu-coordinated complex predominates.

Reaction of Me_2SiCl_2 with [bis(3-phenyl-6-tert-butylinden-1-yl)dimethylsilyl]chlorozirconium tert-butyl amide.

In the glovebox, crude product **3** (30 mg, 0.04 mmol) was dissolved in dichloromethane (1.5 mL) in a 5-mL glass vial equipped with a stir bar. An excess of dichlorodimethylsilane (19 μL, 0.16 mmol) was added at room temperature and the reaction was stirred for 3 h. The solvent was removed under reduced pressure and the product was dried *in vacuo*, affording an orange residue. ^{1}H NMR spectroscopy analysis in CD_2Cl_2 revealed a 4:1 ratio of *rac/meso* of the dichlorozirconocene product,

consistent with the isomer ratio of the initial crude product prior to amide abstraction with Me_2SiCl_2. The co-product $Me_2SiCl[NH$*t*$Bu]$ was also observed.

Attempts towards the *meso*-selective synthesis of 2

According to the procedure reported by Chevalier *et al.* [2], the bis(indenyl) dianion was reacted with *in situ* formed (1-adamantoxy)zirconium trichloride affording complex **4** (Scheme S2). Although in the original contribution, the bulky alkoxy ancillary ligand proved to favour the formation of the *meso* isomer, in our case an equimolar mixture of the two complexes was obtained. Indeed, regeneration of the dichloride species by addition of excess acetyl chloride afforded, along with unreacted proligand, complex **2** as a 1:1 *rac*/*meso* mixture, as observed by ^{1}H NMR spectroscopy on the crude reaction mixture (Figure S6).

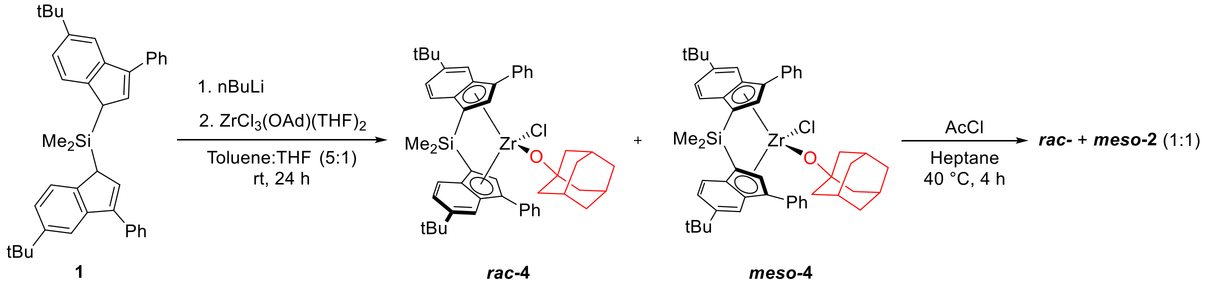

Scheme S2. Attempts towards the *meso*-selective synthesis of complex **2** [2].

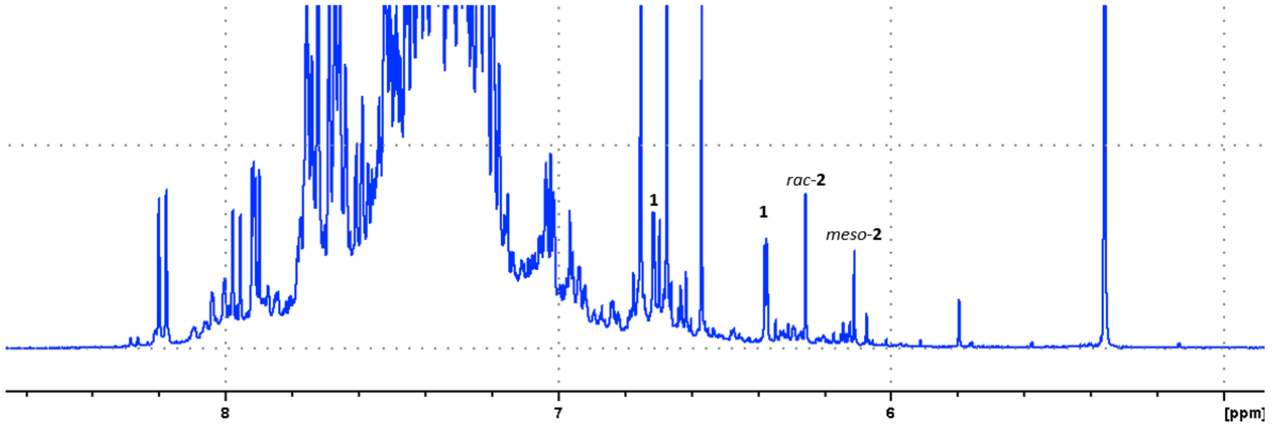

Figure S6. Aromatic region of the ^1H NMR spectrum (400 MHz, CD_2Cl_2, 25 °C) of the crude reaction mixture resulting from the attempted *meso*-selective synthesis of complex **2**, indicating the presence of both *rac*- and *meso* isomers (*ca.* 1:1 ratio) as well as unreacted ligand **1**.

Synthesis of Li_2*-**1** (Solution A)*

Under nitrogen atmosphere, a solution of *n*-BuLi (3.5 mL of a 1.6 M solution in hexanes, 5.56 mmol) was added dropwise at room temperature to a solution of proligand **1** (1.50 g, 2.71 mmol) in a toluene/THF mixture (5:1 *v*/*v*, 6 mL) and the mixture was stirred at room temperature for 4 h (Li_2-**1**, *Solution A*).

Synthesis of $ZrCl_4(THF)_2$ *(Solution B)*

$ZrCl_4$ (629 mg, 2.71 mmol) was suspended in toluene (5 mL). THF (0.45 mL, 5.5 mmol) was added dropwise at room temperature and the mixture stirred at room temperature for 1 h.

Synthesis of Li-OAd (Solution C)

2-adamantol (411 mg, 2.71 mmol) was dissolved in a toluene/THF mixture (5:1 *v*/*v*, 6 mL) and *n*-BuLi (1.86 mL of a 1.6M solution in hexanes, 2.97 mmol) was added dropwise at room temperature; the mixture was then stirred for 1 h.

Synthesis of $Zr(OAd)Cl_3$ *(Solution D).*

Solution C was added by syringe to *Solution B* at room temperature and the mixture was allowed to stir for 2 h ($Zr(OAd)Cl_3$, *Solution D*).

*Synthesis of complex **4** and regeneration of dichloride species **2***

Solution D was added dropwise at room temperature to *Solution A* and the mixture was allowed to stir for 16 h. The solution was concentrated to *ca.* half of its original volume, heptane (10 mL) along with acetyl chloride (0.30 mL, 4.06 mmol, 1.5 equiv) were added and the mixture was then stirred at 40 °C for 4 h. Evaporation of the volatiles afforded a dark-red powder characterized by

^{1}H NMR spectroscopy as a mixture of complex **2** (as a *ca.* 1:1 *rac*/*meso*), unreacted proligand and adamantoxy-derived species.

PE samples characterization

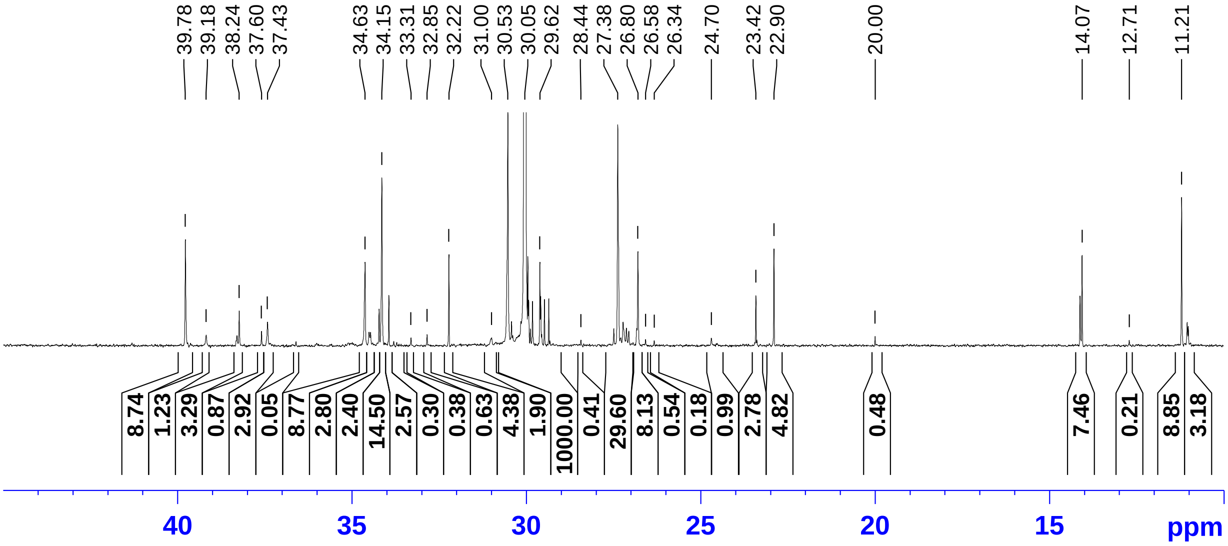

Figure S7. $^{13}C\{^{1}H\}$ NMR spectrum (100 MHz, TCB/C_6D_6, 135 °C) of a PE synthesised with *meso*-**2** in the absence of 1-hexene (Table 1, entry 1).

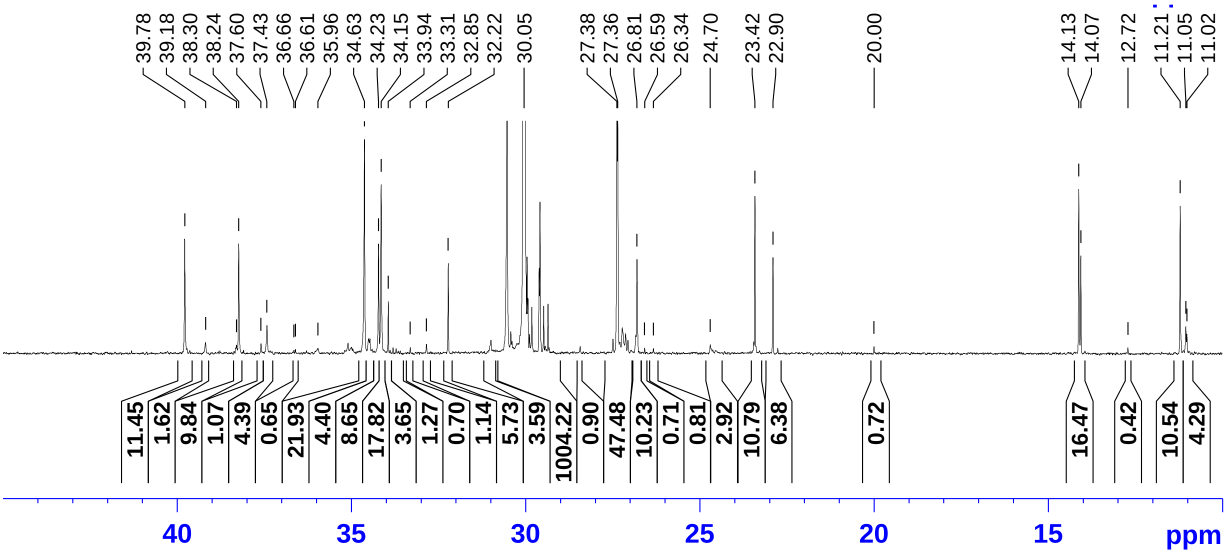

Figure S8. $^{13}C\{^{1}H\}$ NMR spectrum (100 MHz, TCB/C6D6, 135 °C) of a PE copolymer synthesised with *meso*-**2** in the presence 3.0 wt% of 1-hexene (Table 1, entry 13).

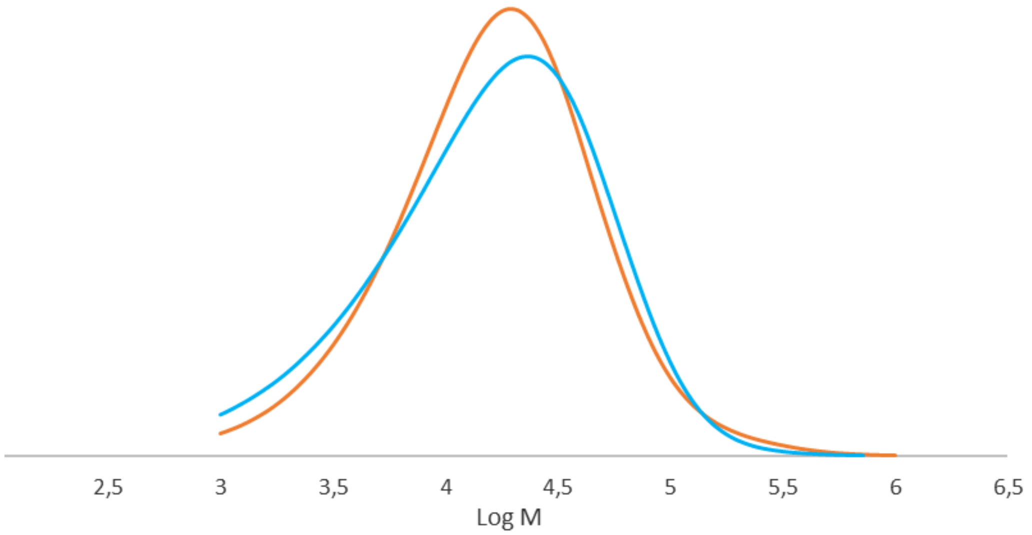

Figure S9. GPC traces of the PEs made with **supp-*rac*-2** (orange) and **supp-*meso*-2** (blue) (Table 2, entries 1 and 2).

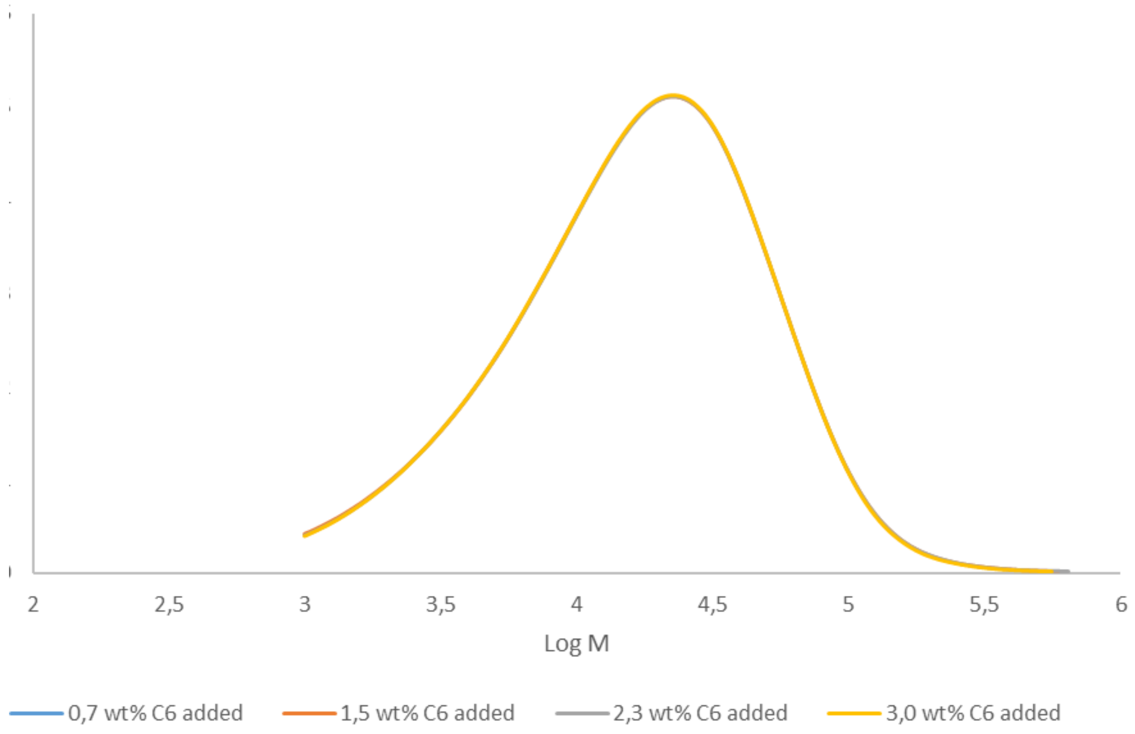

Figure S10. GPC traces of the PEs prepared with **supp-*meso*-2** in the presence of difference amounts of 1-hexene (Table 2, entries 3, 5, 7 and 9): all curves are perfectly overlapped.

Computational Studies.

The calculations were performed using the Gaussian 09 [3] program employing B3PW91 [4,5] functional, and using a standard double-ξ polarized basis set, namely the LANL2DZ set, augmented with a single polarization *f* function on zirconium (0.875) and with a single polarization *d* function on silicon (0.450). The solvent effects, in our case for toluene, were taken into account during all the calculations by means of the SMD model.[6] All stationary points were fully characterized via analytical frequency calculations as either true minima (all positive eigenvalues). Zero-point vibrational energy corrections (ZPVE) were estimated by a frequency calculation at the same level of theory, to be considered for the calculation of the total energy values at T = 298 K in the same way as in the approach used by Castro et *al*.[7]

C	-2.258172	5.458323	0.736119
C	-2.571521	4.026953	2.671052
H	-2.365173	1.910312	2.326786
H	-1.885966	4.447303	-1.133085
H	-2.216286	6.449188	0.290648
H	-2.754892	3.905072	3.735625
H	-2.675406	6.191481	2.732550
C	2.247563	-2.995022	0.586061
C	2.770226	-5.216136	2.270674
C	2.232656	-4.313427	0.075537
C	2.520028	-2.812467	1.960778
C	2.781730	-3.910595	2.791913
C	2.488726	-5.412408	0.907974
H	2.041822	-4.474850	-0.982573
H	2.485617	-1.814803	2.389083
H	2.980870	-3.748873	3.848263
H	2.476223	-6.417404	0.493423
H	2.972171	-6.066679	2.916664
C	5.548627	1.661021	0.941827
C	6.734756	0.668910	0.810351
H	7.590095	1.020553	1.402618
H	7.058170	0.576204	-0.234398
H	6.467980	-0.332273	1.168934
C	5.129255	1.761174	2.430901
H	5.969649	2.126982	3.035887
H	4.824173	0.789324	2.835736
H	4.287775	2.453302	2.556372
C	6.033525	3.053958	0.487586
H	6.871704	3.371589	1.120563
H	5.245927	3.812690	0.579625
H	6.391669	3.049052	-0.550013
C	-5.624307	-1.450104	1.050584
C	-6.176688	-2.835339	0.651677
H	-5.415088	-3.620787	0.738813
H	-6.569870	-2.844598	-0.373045
H	-7.003607	-3.103978	1.321231
C	-5.168650	-1.524919	2.530800
H	-4.808525	-0.556387	2.896586
H	-4.356966	-2.251520	2.656508
H	-6.007984	-1.832212	3.169007
C	-6.772026	-0.414545	0.921258
H	-6.454890	0.586055	1.238397
H	-7.622272	-0.711122	1.549982
H	-7.123570	-0.342118	-0.116078

Cartesian coordinates

rac-2

86

scf done: -1629.580593

Zr	-0.019402	-0.057921	-0.181545
C	1.999027	-1.860027	-0.321448
C	2.717257	-0.602470	-0.374339
C	2.230472	0.139755	-1.522139
C	1.235403	-0.663122	-2.222624
C	1.132262	-1.884563	-1.457388
C	-1.064064	1.785879	-1.522021
C	-1.931446	1.851502	-0.388181
C	-2.714292	0.631107	-0.390739
C	-2.282722	-0.172078	-1.520462
C	-1.242606	0.545357	-2.245916
Si	-0.018453	-0.112427	-3.492341
C	0.573889	1.231208	-4.640488
H	1.494453	0.933845	-5.162086
H	0.765586	2.187769	-4.138798
H	-0.191405	1.422183	-5.407204
C	-0.630200	-1.567854	-4.484941
H	0.115014	-1.820812	-5.253351
H	-1.570624	-1.338738	-5.005104
H	-0.787659	-2.471199	-3.882799
C	3.784296	-0.089982	0.407922
C	2.811613	1.409838	-1.805925
H	2.481714	1.988919	-2.662584
C	4.361399	1.141078	0.103934
C	3.842011	1.883125	-1.009912
H	4.278569	2.845660	-1.253730
C	-2.946085	-1.409348	-1.765916
H	-2.664779	-2.028443	-2.611751
C	-3.794727	0.200547	0.423532
C	-4.438798	-1.007609	0.167618
C	-3.986373	-1.801853	-0.940066
H	-4.483195	-2.742420	-1.152831
H	4.163588	-0.682051	1.234255
H	0.480003	-2.715470	-1.700567
H	-4.126966	0.836313	1.238076
H	-0.369277	2.572065	-1.794556
C	-2.116554	3.025105	0.484894
C	-2.519611	5.314543	2.109405
C	-2.369398	2.895136	1.869197
C	-2.060982	4.325494	-0.066949

C	3.531533	-1.905591	-1.902293
C	4.258691	-2.783223	-2.718161
C	4.751399	-4.172646	-0.790188
H	3.957707	-3.485574	1.093974
H	3.023133	-1.057968	-2.352543
H	4.331372	-2.588343	-3.785128
H	5.221870	-5.049831	-0.352748
H	5.433551	-4.603265	-2.801653
C	-3.339096	-2.198898	-0.532990
C	-4.784260	-3.971886	-2.209430
C	-3.939525	-3.366339	-0.008506
C	-3.471930	-1.941051	-1.916050
C	-4.188377	-2.816248	-2.743908
C	-4.652874	-4.244567	-0.837223
H	-3.863105	-3.575962	1.055575
H	-2.978636	-1.077610	-2.352950
H	-4.268132	-2.605067	-3.807285
H	-5.109025	-5.135561	-0.412724
H	-5.336551	-4.651884	-2.853007
C	-4.102667	3.451790	-1.006920
C	-5.611824	3.097252	-0.953616
H	-6.186695	3.791650	-1.580829
H	-5.997483	3.164352	0.071805
H	-5.804279	2.081381	-1.318230
C	-3.606703	3.354896	-2.473228
H	-4.165466	4.055240	-3.108591
H	-3.743265	2.347692	-2.883751
H	-2.540443	3.601383	-2.545029
C	-3.936408	4.913981	-0.541083
H	-4.518007	5.573263	-1.197752
H	-2.890887	5.244036	-0.588360
H	-4.302953	5.064966	0.482532
C	3.973598	3.571570	-1.060853
C	4.947325	4.390033	-0.172977
H	5.719972	3.744442	0.263771
H	4.428856	4.897736	0.649342
H	5.447694	5.160172	-0.774743
C	4.783400	2.945523	-2.214741
H	4.141392	2.384605	-2.905624
H	5.568479	2.271651	-1.848405
H	5.274288	3.739189	-2.792022
C	2.914459	4.524895	-1.674094
Cl	0.832278	1.472529	1.491651
Cl	-0.949156	-1.711658	1.321330

meso-2

86

scf done: -1629.582374

Zr	0.021963	-0.655390	0.207033
C	-2.603170	-1.289302	0.363884
C	-2.663297	0.159088	0.391759
C	-1.889654	0.612906	1.531199
C	-1.364617	-0.547556	2.244626
C	-1.845612	-1.691037	1.507625
C	1.893638	0.667667	1.508857
C	2.675211	0.214615	0.374512
C	2.655739	-1.233278	0.370368
C	1.910358	-1.635446	1.522238
C	1.407334	-0.494611	2.246793
Si	0.021618	-0.634866	3.488246
C	-0.006460	0.694694	4.795006
H	-0.943756	0.644610	5.367658
H	0.087111	1.715349	4.406795
H	0.821942	0.541468	5.501778
C	0.050190	-2.312609	4.302660
H	-0.822407	-2.434382	4.960184
H	0.950962	-2.426630	4.922266
H	0.037339	-3.145027	3.587756
C	-3.367422	1.091710	-0.412625
C	-1.830731	2.009833	1.798020
H	-1.286723	2.382906	2.659772
C	-3.312159	2.455870	-0.132081
C	-2.523595	2.892437	0.984303
H	-2.477092	3.950660	1.218165
C	1.796774	2.071941	1.746181
H	1.238032	2.452674	2.594747
C	3.353472	1.149743	-0.455750
C	3.260817	2.512282	-0.202151
C	2.462854	2.950879	0.912962
H	2.389575	4.015450	1.118957
H	-3.968293	0.728066	-1.239784
H	-1.652692	-2.725102	1.767963
H	3.956581	0.782617	-1.277042
H	1.744330	-2.669980	1.798970
C	3.407202	-2.142384	-0.514747
C	4.873798	-3.920819	-2.167174
C	4.027674	-3.291690	0.026473

H	2.207595	3.971207	-2.303749
H	3.406764	5.285657	-2.294306
H	2.336866	5.047706	-0.902035
Cl	-0.007204	0.968159	-1.591166
Cl	0.041251	-2.671147	-1.131220

References

[1] a) Nifant'ev, I. E.; Ivchenko, P. V.; Bagrov. V. V.; Churakov, A. V.; Chevalier, R. *Organometallics* **2012**, *31*, 4340-4348. b) Nifant'ev, I. E.; Ivchenko, P. V.; Bagrov. V. V.; Churakov, A. V.; Mercandelli, P. *Organometallics* **2012**, *31*, 4962–4970.

[2] Chevalier, R.; Garcia, V; Müller, P.; Sidot, C.; Tellier, C.; Delacray, L. WO2005058929A1.

[3]. Frisch, M. J.; Trucks, G. W.; Schlegel, H. B.; Scuseria, G. E.; Robb, M. A.; Cheeseman, J. R.; Scalmani, G.; Barone, V.; Mennucci, B.; Petersson, G. A.; Nakatsuji, H.; Caricato, M.; Li, X.; Hratchian, H. P.; Izmaylov, A. F.; Bloino, J.; Zheng, G.; Sonnenberg, J. L.; Hada, M.; Ehara, M.; Toyota, K.; Fukuda, R.; Hasegawa, J.; Ishida, M.; Nakajima, T.; Honda, Y.; Kitao, O.; Nakai, H.; Vreven, T.; Montgomery, J. A., Jr.; Peralta, J. E.; Ogliaro, F.; Bearpark, M.; Heyd, J. J.; Brothers, E.; Kudin, K. N.; Staroverov, V. N.; Kobayashi, R.; Normand, J.; Raghavachari, K.; Rendell, A.; Burant, J. C.; Iyengar, S. S.; Tomasi, J.; Cossi, M.; Rega, N.; Millam, J. M.; Klene, M.; Knox, J. E.; Cross, J. B.; Bakken, V.; Adamo, C.; Jaramillo, J.; Gomperts, R.; Stratmann, R. E.; Yazyev, O.; Austin, A. J.; Cammi, R.; Pomelli, C.; Ochterski, J. W.; Martin, R. L.; Morokuma, K.; Zakrzewski, V. G.; Voth, G. A.; Salvador, P.; Dannenberg, J. J.; Dapprich, S.; Daniels, A. D.; Farkas, Ö.; Foresman, J. B.; Ortiz, J. V.; Cioslowski, J.; Fox, D. J. Gaussian 09, Revision D.01; Gaussian Inc.: Pittsburgh, PA, 2009.

[4] Becke, A. D. *Phys. Rev. A* **1988**, *38*, 3098–3100.

[5] Becke, A. D. *J. Chem. Phys.* **1993**, *98*, 5648–5652.

[6] Marenich, A. V.; Cramer, C. J.; Truhlar, *J. Phys. Chem. B*, **2009**, *113*, 6378–6396.

[7] Castro, L.; Kirillov, E.; Miserque, O.; Welle, A.; Haspeslagh, L.; Carpentier, J.-F.; Maron, L. *ACS Catal.* **2015**, *5*, 416–425.
